# Technology Acceptance and Quality of Life among Older People Using a TUI Application

**DOI:** 10.3390/ijerph16234706

**Published:** 2019-11-26

**Authors:** Way Kiat Bong, Astrid Bergland, Weiqin Chen

**Affiliations:** 1 Department of Computer Science, OsloMet – Oslo Metropolitan University, Postboks 4, St. Olavs plass, 0130 Oslo, Norway; Weiqin.Chen@oslomet.no; 2 Department of Physiotherapy, OsloMet – Oslo Metropolitan University, Postboks 4, St. Olavs plass, 0130 Oslo, Norway; astridb@oslomet.no

**Keywords:** older people, tangible user interface, technology acceptance model, quality of life

## Abstract

Good quality of life is important for healthy ageing. Studies have shown that although information and communication technology can improve older people’s quality of life, their technology acceptance level is rather low. Tangible user interfaces (TUIs) enable people to interact with the digital world through everyday physical objects, thus offering more intuitive digital environments for older people. In this study, we employ a TUI prototype to investigate the relationship between older people’s technology acceptance and quality of life, the changes in these outcome measures after using TUI, and the associations between them. The TUI prototype, Tangible Cup was used by 20 older participants over a period of three months. Data were collected using the technology acceptance model (TAM) questionnaire, the older people’s quality of life (OPQOL) questionnaire and semi-structured interviews. The results showed some positive changes in technology acceptance after the use of Tangible Cup. However, no change in the quality of life was found. While statistically significant correlations between the change in technology acceptance and the change in quality of life were observed, limitations such as small sample size and participants not accurately representing the target population should be noted. Thus, further research is needed to better understand the associations between the change in technology acceptance and the change in quality of life.

## 1. Introduction

Quality of life is reportedly strongly associated with health in older people. A cohort study by Iwasa et al. [1] and meta-analyses [2] have reported that people who find their lives worth living have a lower risk of mortality and cardiovascular diseases compared to those who do not. According to the World Health Organization (WHO), quality of life refers to individuals’ perceptions of their position in life in the context of the culture and value systems in which they live, and in relation to their goals, expectations, standards, and concerns [3]. Bowling [4] and Bowling et al. [5] stated that there is international interest in enhancing and measuring the quality of life in older age partly because of the increasing number of older people and higher expectations of life within the society. Quality of life is a useful concept in this context; one that shifts our perspective from a narrow medical definition of health to one encompassing the broader aspects of well-being recognized by older people themselves [6].

Studies have shown that the use of information and communication technologies (ICT) could contribute to improving older people’s well-being and quality of life [7,8,9,10]. Assistive technology in older people’s care, such as video-monitoring, remote health monitoring, fall detectors, pressure mats and other electronic sensors and equipment were identified by Miskelly [11] as having the potential to make an important contribution to the care of older people as long as they fit the person’s needs and lifestyle. However, the older people who could benefit most from using digital technology are not usually the ones using it [12]. 

Because of the difficulties in learning and using ICT, and the belief that ICT is not necessary in their daily life, older people have low acceptance and use. As previous research on technology acceptance has highlighted, many technological interventions could be perceived as a waste of time and money because people do not fully accept and use the technology [13]. Several studies have been conducted to improve the older people’s acceptance and use of ICT [8,14,15,16]. Fischer et al. [17] identified barriers faced by the older people in accepting and using health information technology and they highlighted the importance of designing new technology with the needs of older people in mind. Neves and Amaro [18] studied the use and perception of ICT among older people in Lisbon, Portugal, and found that the lack of functional literacy in ICT was their main reason not to use a computer or the Internet. Chou et al. [19] have studied technology acceptance and quality of life among older people in a telecare programme in Taiwan. They found a strong association between these two outcome measures. Older people who used the telecare programme frequently had better social welfare status, and they scored higher in their technology acceptance and quality of life. 

Tangible user interface (TUI) is a form of user interface that couples digital information with everyday physical objects and architectural surfaces [20]. The aim is to enhance the interaction between humans and digital information. TUI has been developed for older people with the potential to improve their technology acceptance [21,22] and quality of life [23]. Spreicer [22], Davidoff, Bloomberg, Li, Mankoff, and Fussell [21] designed TUI applications for older people to send email or short message service (SMS) to each other. Marques, Nunes, Silva, and Rodrigues [23] aimed to improve the quality of life of older adults by using TUI to provide a better and richer digital game experience. Despite TUI’s potential to provide an intuitive interface and better ICT experience for older people, very little research has been conducted on the impact of TUI on older people’s technology acceptance and on their quality of life as a whole.

In this study, we aim to investigate the relationship between older people’s technology acceptance and quality of life, the changes in these two after using a TUI intervention, and the association between the changes in technology acceptance and the changes in quality of life. We focus on older people living alone at home who may be impacted in terms of their technology acceptance and quality of life after using the TUI intervention. The technology acceptance model (TAM) questionnaire and the older people’s quality of life (OPQOL) questionnaire were used to measure their technology acceptance and quality of life. We provided a TUI prototype, Tangible Cup to 20 older participants and asked them to use it for three months. 

## 2. Materials and Methods

### 2.1. Participants

A total of 20 older people (18 women and 2 men, aged 72–89 years) were recruited. They were recruited through a previous project related to quality of life, nutritional status, physical condition and pain, mental and social function among senior center users. The potential participants were first identified and then contacted to be briefed about this study. Our inclusion criteria were that they lived alone, were over 70 years, and were able to walk independently with or without an assistive device indoors. However, as we had problems recruiting male participants, we decided to recruit one man who was living with his wife. 

### 2.2. Ethical Considerations

Our study was pre-approved and registered by the Norwegian Centre for Research Data (NSD); reference number 253545. Prior to participating in our study, the participants were briefed with written and oral information about the study. After receiving the information, all the participants gave their informed consent. This included the assurance that they could withdraw their consent without consequences at any time. 

### 2.3. The Prototype—Tangible Cup

Tangible Cup is inspired by the idea of adopting TUI to encourage social interaction among older people [24]. The main function of the Tangible Cup is to connect older users to new potential friends. The users did not know each other when they started using the Tangible Cup and they were supposed to make calls to the other online users listed on an app on a tablet. 

We have previously designed and developed Tangible Cup by using a user-centered design [25] and a co-design approach [26]. Four iterations of design, implementation, and usability testing were conducted and two older participants were asked to perform a series of testing tasks in the usability testing. 

**Figure 1:**
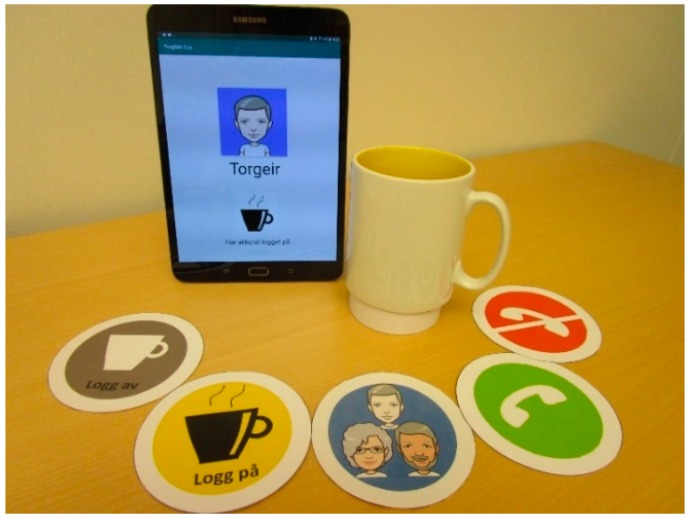
Tangible Cup.

Figure 1 illustrates the components of a Tangible Cup set, which consists of a cup attachment (under the cup), five cup coasters (from left to right: *log out, log in, search contacts, call* and *end call*), and a tablet. To use Tangible Cup, the users moved and placed the cup attachment on the respective cup coasters to perform the tasks. For instance, placing the cup attachment on the *log in* cup coaster will start the calling app in the tablet and log the user in.

### 2.4. Instruments

This study adopts a mixed qualitative and quantitative methods approach. The OPQOL questionnaire and TAM questionnaire were used to collect quantitative data while semi-structured interviews were conducted to collect qualitative data.

#### 2.4.1. Technology Acceptance Model (TAM)

To develop our TAM questionnaire, we referred to other existing studies that had used the TAM questionnaire to investigate older people’s technology acceptance [27,28,29,30,31], and adapted the questions to reflect the use and acceptance of TUI. From these studies, eight determinants were identified as related to technology acceptance for the use of TUI. The determinants are (a) perceived usefulness, (b) perceived ease of use, (c) perceived enjoyment, (d) intention of use, (e) actual use, (f) compatibility, (g) attitude, and (h) self-efficacy. Likert scales from 1 to 7 (1 is strongly disagree and 7 is strongly agree) were used to evaluate the statements related to participants’ use of ICT tools. The questionnaire items are presented in Table 1.

#### 2.4.2. Older People’s Quality of Life (OPQOL)

The OPQOL questionnaire was developed by Bowling [32] as a new measure of quality of life in older age. Using Likert scales from 1 to 5 (1 is strongly agree and 5 is strongly disagree), it evaluates the quality of life of older adults in eight dimensions, i.e., (a) life overall, (b) health, (c) social relationships and participation, (d) independence, control over life, freedom, (e) home and neighborhood, (f) psychological and emotion well-being, (g) financial circumstances, and (h) leisure and activities. Each dimension has four to six questions. The questionnaire was generated based on older people’s responses on the positive aspects that contributed to a good life, and negative aspects that reduce their quality of life. The first question, “Thinking about both the good and bad things that make up your quality of life, how would you rate the quality of your life as a whole,” evaluates the respondent’s quality of life as a whole from very good (1) to very bad (5). The remaining 35 questions are statements, which respondents answer by selecting alternatives from strongly agree (1) to strongly disagree (5).

We translated the OPQOL questionnaire into Norwegian based on the guidelines developed by Beaton et al. [33]. The original OPQOL questionnaire in English was first translated into Norwegian by two native Norwegians. The translated OPQOL questionnaire was then back translated from Norwegian into English by an English native translator. Lastly, the two Norwegian natives went through the translated OPQOL questionnaire from the English native translator and approved the translation.

#### 2.4.3. Semi-Structured Interview

A semi-structured interview guide was used and follow-up questions were asked to clarify their answers. The aim is to gain deeper insight into their experience of using Tangible Cup and to explore the potential of Tangible Cup. Examples of interview questions were: “Tell me about your experience of using ICT/the Tangible Cup?,” “Do you have any problems using the Tangible Cup?,” “When/How often do you use the Tangible Cup?,” “What do you think of the conversations that you have had?,” “How do you feel after using the Tangible Cup?,” and “Anything positive/negative about the Tangible Cup?” Each interview lasted less than an hour and was conducted at the participant’s home with only the interviewer and the participant present.

### 2.5. Data Collection

**Figure 2:**
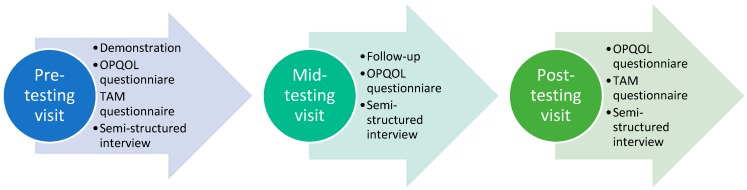
Visualization of data collection process.

We performed the data collection for OPQOL and semi-structured interview three times, i.e., pre-testing, mid-testing, and post-testing, while we performed TAM twice, i.e., pre-testing and post-testing. The overall data collection process is summarized in Figure 2. Informed consent was given prior to participating in the longitudinal study. The participants were given a Tangible Cup set during the first visit to their home. They were given a demonstration of how to use Tangible Cup and asked to use it whenever they liked. They then filled out the OPQOL and TAM questionnaires, and were interviewed. 

After one and a half months, we visited the participants for the second time. Some of the participants had yet to start using their Tangible Cup. Therefore, during the mid-testing visit TAM questionnaire was not filled out and only the OPQOL questionnaire was answered. The participants were then interviewed about their experience of using Tangible Cup. 

After another one and a half months, we conducted the post-testing visit. We asked the participants to fill out both the OPQOL and TAM questionnaires, and then conducted a semi-structured interview. 

### 2.6. Data Analysis

The OPQOL and technology acceptance scores, and the changes in these two by dimensions were analyzed. The OPQOL scores are originally 1 to 5 for possible options “strongly agree” and “very good” to “strongly disagree” and “very bad” for 35 statements evaluating the quality of life from different dimensions, and for the first question rating overall quality of life. We transformed and computed all the scores so that the higher scores indicate better quality of life. The scores were summed up by dimensions, i.e., (D1) life overall, (D2) health, (D3) social relationships, and participation, (D4) independence, control over life, freedom, (D5) home and neighborhood, (D6) psychological and emotion well-being, (D7) financial circumstances, and (D8) leisure and activities. 

In the TAM questionnaire, scores range from 1 to 7 for possible options “strongly disagree” to “strongly agree” for 21 statements. The scores were summed up by dimensions, i.e., (D1) perceived usefulness, (D2) perceived ease of use, (D3) perceived enjoyment, (D4) intention of use, (D5) actual use, (D6) compatibility, (D7) attitude, and (D8) self-efficacy. The higher scores indicate higher technology acceptance.

The outcome variables (change in technology acceptance and OPQOL) were described using median. To assess the possible associations between OPQOL and technology acceptance, we computed Spearman’s correlation coefficients [34]. First, we calculated the correlation between technology acceptance and OPQOL at baseline. Second, we measured both outcomes again at post-testing and computed a correlation between changes in technology acceptance and changes in OPQOL. All tests were two-sided and values of *p* < 0.05 were considered statistically significant. As our study was considered an exploratory analysis, no correction for multiple testing was applied. All analyses were performed using the SPSS version 25. 

Inductive content analysis was performed to analyze the qualitative data. This approach is suitable when the study is explorative or there are no existing studies in the research field [35,36]. Our study meets both criteria.

The interviews were firstly transcribed. The transcript was then read and analyzed in three main steps, i.e., open coding, creating categories, and abstraction [35]. During open coding, notes and headings were written down while reading the transcript. The next step was creating categories. These notes were grouped into categories to increase our understanding of the participants’ use of Tangible Cup [37]. These categories helped us to describe participants’ experience of using Tangible Cup, which links the impacts of using the Tangible Cup to the participants’ technology acceptance and quality of life. Lastly, we performed abstraction to formulate the generated categories from the previous step into a main category. NViVo 12 was used to perform the three-step inductive content analysis process.

## 3. Results

### 3.1. Characteristics of Participants

Table 2 summarizes the characteristics of the participants. During our visits to the participants, they were observed on how they used their smartphone and/or tablet. Their ICT skills level was assessed based on these observations. Four participants (P17, P18, P19, and P20) decided to withdraw from the study after one month. Their data from the pre-testing were included in the data analysis and results. 

### 3.2. OPQOL Questionnaire and TAM Questionnaire

The correlations between the total score of OPQOL and all the OPQOL dimensions with the technology acceptance total score before using Tangible Cup are presented in Table 3. No statistically significant correlation was found. By showing the correlation between pre-testing OPQOL and TAM, it can then assume that the correlation observed in post-testing is due to the use of Tangible Cup.

In terms of the changes in technology acceptance scores among participants from pre-testing to post-testing, 12 participants scored higher after using Tangible Cup, while four participants (P9, P13, P14, and P16) scored lower. 

**Figure 3:**
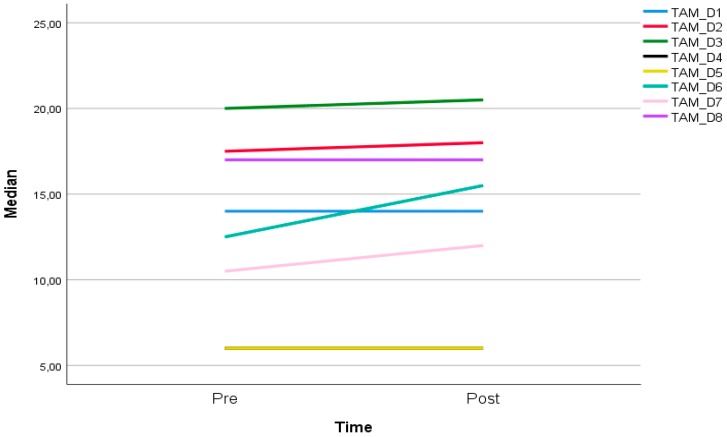
Median score of technology acceptance by dimensions at pre and post testing.

Referring to Figure 3, in terms of dimensions in technology acceptance, with the exception of D1 (perceived usefulness), D4 (intention of use), D5 (actual use), and D8 (self-efficacy), there were increments in all dimensions. D6 (compatibility) increased the most, followed by D7 (attitude), D2 (perceived ease of use), and D3 (perceived enjoyment). 

To study the changes in scales by dimensions, we summed up the changes from pre-testing to post-testing in negative scale, i.e., scale 1, 2, and 3 (Strongly disagree, Disagree, Somewhat disagree), and positive scale, i.e., scale 5, 6, and 7 (Somewhat agree, Agree and Strongly agree). Referring to Table 4, we can see that D6 (compatibility) and D7 (attitude) show the most improvement. D6 (compatibility) had an increase of 18.76% in positive scale (Scale 5, 6, and 7) and a decrease of 18.76% in negative scale (Scale 1, 2, and 3). D7 (attitude) has a 21.86% increase in positive scale and a 9.38% decrease in negative scale. 

When we look at individual questions, we can see that there were greater changes in some questions (Q2, Q4, Q5, Q6, Q7, Q8, Q9, Q11, Q12, Q13, Q14, Q15, Q16, Q18, Q20, and Q21) than in others. Q4, Q5, Q7 (D2—perceived ease of use) and 8, 9, and 11 (D3—perceived enjoyment) had two individual scales with changes of more than 18%, while the others had one. This indicates that the use of Tangible Cup had the greatest impact on these two dimensions. When we studied the changes in positive scale and negative scale as a whole, the greatest improvement was in Q7 (D2—perceived ease of use), Q15 and Q16 (D6—compatibility) while the largest decrease was in Q10 (D3—perceived enjoyment). Nine out of 16 participants gave higher scores when they were asked whether it is easy to learn to use digital communication tools (Q7). Half of the participants were more positive about the use of digital communication tools fitting their lifestyle (Q15) and their way of socializing with others (Q16). Table 4 summarizes all the scores by scale, question, and dimension in percentage.

In terms of the changes in OPQOL, none of the participants’ OPQOL scores improved all the way from pre-testing to post-testing. Eight out of 16 participants had improved their OPQOL score at the mid-study, but the OPQOL scores decreased again at post-testing. The other eight participants’ OPQOL score decreased at mid-testing. However, six of these eight participants scored higher at post-testing. There was no change in one participant’s score while the other participants’ OPQOL scores continued to fall. While studying the changes in the total scores of OPQOL, it is essential to mention that the use of Tangible Cup was believed to impact on some dimensions of the participants’ OPQOL, but not on all of them, for instance, D7—financial circumstances.

**Figure 4:**
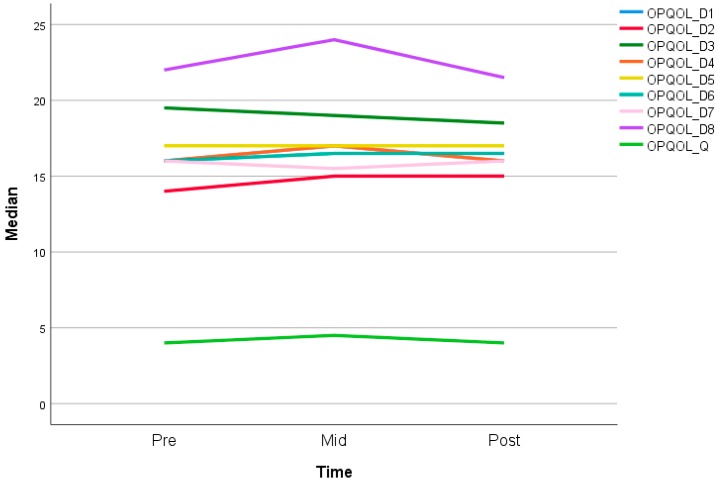
Median score of OPQOL by dimensions for pre, mid and post testing.

Figure 4 illustrates the change in median score of OPQOL by dimensions (D1–D8) and Q (the first question in the questionnaire about the participants’ quality of life as a whole) from pre-testing to mid and post-testing. D3 (social relationships and participation) is the only dimension showing negative correlation over time. Although there are changes in Q (overall quality of life), D1 (life overall), D4 (independence, control over life, freedom), and D7 (financial circumstances) from pre-testing to mid-testing, their median score remains the same when we compare pre-testing to post-testing. D2 (health) and D6 (psychological and emotion well-being) scored higher at the end of the study while D8 (leisure and activities) scored lower. There was no change in D5 (home and neighborhood) during the study. 

The results of Spearman’s rank-order correlation [34] are summarized in Table 5. Our data reveal some relevant statistically significant correlations. D5 in OPQOL (home and neighborhood) has a significant positive correlation with overall technology acceptance (TAM), D2 (perceived ease of use) and D8 (self-efficacy) in TAM. While Q of OPQOL (first question accessing overall quality of life) is negatively correlated with D5 (actual use), it is nonetheless positively correlated with D7 (attitude) in TAM. D4 in TAM (intention of use) is also positively correlated with OPQOL (total score) and D7 in OPQOL (financial circumstances). However, as mentioned earlier, the use of Tangible Cup is not expected to have any impact on the participants’ financial circumstances. 

### 3.3. Semi-Structured Interview

**Figure 5:**
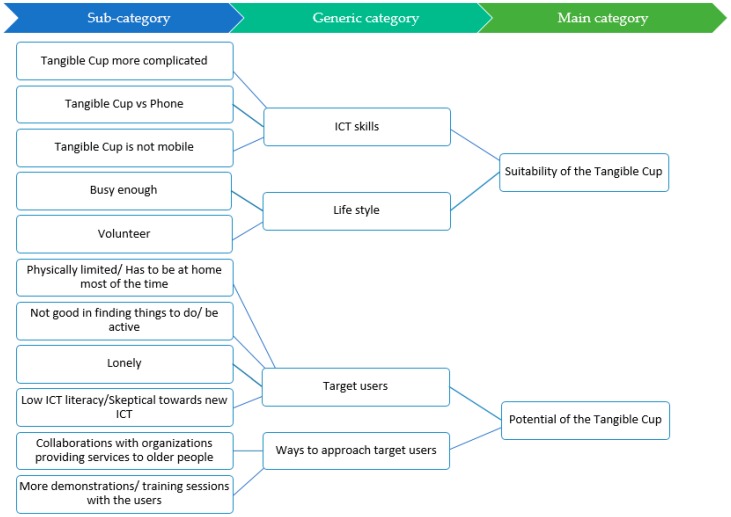
The abstraction process with sub-categories, generic categories and main categories

Using the three-step inductive content analysis, two main categories, i.e., “suitability of the Tangible Cup” and “potential of the Tangible Cup,” were generated. Figure 5 illustrates the abstraction process in our content analysis and the generated categories. 

#### 3.3.1. Suitability of the Tangible Cup

Four participants who withdrew from the study (P17, P18, P19, P20), provided the reasons that the Tangible Cup was not suitable for them. After using Tangible Cup for a month, they did not see the need and benefit of using the Tangible Cup and one mentioned the lack of male participants.

The participants have different levels of ICT skills (refer Table 2) and TUI might not be suitable for them. More than half of them are advanced IT users. Although their use of ICT is not entirely error and problem-free, they do use a smartphone on a daily basis. Some of them even use a tablet regularly. They therefore perceived the Tangible Cup as being more complicated as they could already use a touch screen without much difficulty. Out of a total of 16 remaining participants, seven switched from using the cup attachment as their TUI object to only using the tablet without a TUI object after our mid-study visit. They commented that without the cup attachment, it was easier to use the calling app with touch gestures on the tablet. Only four participants continued to only use the cup attachment throughout the study while the rest of them switched between using and not using the cup attachment.

*“I liked it better when I was informed that I could use the tablet without these cups. I think so. Because then I only had to concentrate on one thing, so it was easier for me.”*

Since the participants have had experience of using smart phones and tablets, they tended to expect the Tangible Cup to work like the devices that they were used to. This expectation caused some usability challenges during their three-month use of Tangible Cup. For instance, some of them misunderstood and thought the Tangible Cup worked like a phone. They expected the other users being called to hear the app ringing and answer their call immediately.

*“I made a call to one person here, and it rang and rang, and no one picked up the phone. Then I tried two more, the same day! After that I sent a SMS to you (referring to the main author of this paper). I was quite irritated, that I had to sit here and waste my time on this thing!”*

The Tangible Cup is an Internet-based app and there were other external factors that influenced the ways the Tangible Cup app was used. For instance, we observed that some users did not log out properly after using the Tangible Cup. They thought that turning off the tablet’s screen made them log out of the app. However, the app was actually still running. This resulted in many unanswered calls because the logged on users were not actually present.

Furthermore, the participants perceived the Tangible Cup as not being mobile. Although the whole Tangible Cup set is not heavy and easy to bring around, many users only used it at a certain place. They had the expectation that the Tangible Cup should be mobile like their smart phones.

*“You have to sit down here with this thing and have it in front of you. But a phone is something you can have in your pocket and answer. You don’t need to sit down here to deal with it, you can do it at the kitchen table, or in the bathroom or anywhere. You can even sit on the toilet and talk on the phone, right? You can’t do that with this thing (Tangible Cup) here.”*

Lastly, some of the participants expressed that they were already busy enough on a daily basis. Many of them participated in our study because they wanted to help other older people who might feel lonely and need someone to talk to. This resulted in too few users online at the same time. We therefore arranged two time slots which the participants should try to use Tangible Cup, i.e. 3 pm to 5 pm and 7.30 pm to 9.30 pm.

*“I go to the gym, meet friends, take care of the grandchildren….So I’m actually doing something all the time. So I don’t always remember this (referring to the Tangible Cup) is laying there. And since it isn’t ringing, I don’t do anything with it. If it rang then I would pick it up, if you understand? But, that’s how it is…”*

#### 3.3.2. Potential of the Tangible Cup

Although most of the participants were not the target user group for the Tangible Cup, they recognized its potential. All of the participants agreed that the Tangible Cup had the potential to make a great impact if it could reach the target user group and all the features could be properly and fully developed. The primary characteristic of the target group is older people with limited physical ability or whose movement is restricted, which means they have to stay at home most of the time. Some of them are not good at finding things to do. So, while they cannot go out and make new friends, the Tangible Cup offers them the possibility to do that at home.

*“I do know people who sit alone at home the whole winter, because it’s so slippery right? And they do become very lonely at home by themselves. Because their friends might not be able to go out either. So then it is quite a crisis for them, some people I know.”*

*“I have an uncle who is 95 years old. He is bad with his feet, but his mind is totally fine. So my uncle in Drammen could certainly have enjoyed a system like this.”*

The Tangible Cup could be a great help to older people who feel lonely. Some participants mentioned that as they become older, there are fewer people in their social circle. So for those who are getting older and older and feel lonely at times, the Tangible Cup can help them to make new friends.

*“It would certainly be suitable for very lonely people too, but there has to be two people. So one of them could be very lonely, and the other could be relatively healthy and active. It will be a combination where one person doesn’t have much going on and can then call the other one.”*

In addition, the Tangible Cup may be suitable for older people with low ICT skills or who are skeptical to new technology. When one becomes older, one might suffer from memory decline and therefore, older people become more forgetful [38,39]. The use of cup coasters and a cup attachment to control the app in the tablet was regarded as easy and required less effort to remember, and could thus be an easier approach for non-native older ICT users.

*“If you are in a phase where you can easily select someone you know, and you don’t have to think about anything other than that cup and those cup coasters, because the rest sorts itself out, right. So I see the point, I do.”*

In order to reach the right target user group in future, we need to collaborate with organizations that have experience of providing services to older people. The older users who use their services trust them and have faith in them. In addition, these organizations shall also become users of Tangible Cup. The older users can reach them easily by using Tangible Cup.

*“I think the idea is good, but one has to find a way to use it. I think there’s certainly many people sitting alone (at home), and they would then have someone to call, three, four, five people to call. Seeing the names displayed there (referring to Tangible Cup), when they’re logged in. So I think it can be useful, something like a social service, absolutely… I think.”*

The use of Tangible Cup can be extended from homes to places such as senior centers and community centers. More demonstrations or training sessions could be held with users at their local senior centers to encourage older people to use it. They can learn how to use Tangible Cup as a group, and use it there regularly with others.

*“We also do that at the senior center, so there are more people working with it. But most of them are only in one place, not at someone’s home. When one is using it alone (at home), one loses courage quickly, one does that.”*

Arrangements can be made to enable people to be logged in to Tangible Cup with other users at other senior centers at the same time. The idea is similar to the sessions we have previously arranged for the participants. It has proven useful to get the users online at the same time.

## 4. Discussion

To the best of our knowledge, no previous research has been conducted on the use of TUI as an intervention to study its impact on older people’s technology acceptance and quality of life, and the associations between these two outcome measures. We have presented the quantitative and qualitative results in the previous section. In this section, we present the interpretation of our results after analyzing both the qualitative and quantitative data as a whole. 

### 4.1. Impact of TUI on Older People’s Technology Acceptance

The study shows that 12 out of 16 participants’ technology acceptance improved after using Tangible Cup. Similar to Davidoff, Bloomberg, Li, Mankoff and Fussell [21]’s and Spreicer [22]’s findings, TUI has the potential to increase older people’s confidence in using new ICT, especially for those with low ICT literacy. Chen and Schulz [40] drew the conclusion that ICT is not a one-solution-for-all with respect to older people, who make up a large and diverse population [24,41]. They can be very different from one another when it comes to their preferences, abilities, demographic background, social status etc. A single TUI application such as the Tangible Cup is not therefore necessarily suitable for all older people. This is demonstrated by the scores in D1 (perceived usefulness), D4 (intention of use), and D5 (actual use) in our study. D1 (perceived usefulness), D4 (intention of use), D5 (actual use) indicate that the use of Tangible Cup might not be suitable for the participants. P12-P16 are among the participants that scored the lowest improvement in these three dimensions, and they are all advanced ICT users. Three out of four participants (P13, P14, and P16) who scored lower in technology acceptance after using Tangible Cup are also advanced ICT users. They found TUI a more challenging interface to use as they already mastered the use of touch screen. The same goes for P17 (advanced) and P19 (very advanced) who withdrew from our study, as they did not find the Tangible Cup useful as they could already perform all their social interaction using their touch screen smart phones and tablets. 

D7 (attitude) is one of the dimensions that shows the most improvement. The scores for Q17 and Q18 (D7 in TAM), together with the positive feedback from the participants about the potential of Tangible Cup in the semi-structured interviews, indicate that the use of Tangible Cup can improve older people’s attitudes to using new ICT. Mitzner et al. [42] reported that most older people are positive, rather than negative, in accepting technology, as long as the technology does not cause inconvenience, harms their security, or is unhelpful. Despite the challenges they faced in using Tangible Cup, the participants managed to see the potential of Tangible Cup, which could possibly explain their higher score in D7 (attitude) in TAM.

D6 (compatibility) in TAM had the biggest increase in its median after the study, and all three questions in D6 (Q14, 15, 16) had significant positive changes. Compatibility refers to the way users value a product, and how the product fits their needs and lifestyle [43]. Tangible Cup, which was inspired by the Norwegian coffee-drinking culture [24], fits well with the participants’ lifestyle (Q15) and has the potential to work well as a communication tool (Q16) fitting well with their social life (Q14). Nevertheless, similar to the finding of a study investigating older people’s participation in video user-created content (video UCC) [44], the increase in compatibility (D6 in TAM) has neither increased the participants’ intention to use nor actual use in technology acceptance (D4 and D5 in TAM). Both the results of the semi-structured interviews and the TAM questionnaire indicate the same, i.e., that the participants did not fit exactly into the Tangible Cup target user group. The participants highlighted the usability challenges in terms of using Tangible Cup, as they were already familiar with using touch gestures on their smartphones and tablets. Thus, they did not use their Tangible Cup very often, and did not improve much with respect to their actual use (D5, Q13) in TAM. 

However, all of the participants agreed in the interviews that the Tangible Cup could be useful and beneficial to a certain target user group, which includes those who need new friends, have low ICT literacy and probably restricted physical movement as well as a need for ICT training courses. Blažun et al. [45] concluded that using ICT as a means of encouraging social and physical activities among older people is promising. Their findings show that older people who are less ICT literate and socially and physically active have a higher chance of benefitting from the positive effects of adopting ICT. Tangible Cup is probably suitable for older people with these characteristics. 

### 4.2. Impact of TUI on Quality of Life

Our results showed that there were changes in the participants’ quality of life, but none of them are statistically significant. The concept of “quality of life” is broad and yet complex, and affected by a person’s physical health, psychological state, level of independence, social relations, personal beliefs, and relationship to the environment [3]. In order to provide an accurate instrument to measure older people’s quality of life, Bowling [32] has specifically developed the OPQOL questionnaire, which was used in our study. This questionnaire has been used in studies predicting adverse health outcomes [46], investigating the associations between frailty and quality of life [47], etc. However, it has not been used in any studies using ICT as an intervention. We acknowledge that the use of Tangible Cup could probably only affects certain aspects of the participants’ quality of life, i.e., social relationships and participation, and psychological and emotion well-being.

The analysis of OPQOL data shows that the median score of D3 (social relationships and participation) in OPQOL decreased consistently from pre-testing to post-testing. This decline can be explained using the results of the semi-structured interviews. Most of the drop in the participants’ D3 scores was probably due to the challenges they faced in using Tangible Cup outweighing the social relationships and participation they had gained. In the semi-structured interviews, many of them voiced their frustration about not getting an answer when they called someone, not being able to bring the Tangible Cup around etc. Some of the participants had high hopes and expectations when they started using Tangible Cup. However, because of these frustrations, they were disappointed by the use of Tangible Cup and this resulted in a lower score in D3. All of them scored lower in Q10 in the TAM questionnaire after the study, as they found it less pleasant to use ICT tools after using Tangible Cup. This could correspond with Dickson and Gregor [41]’s argument that the positive effects of using computer systems among older people can be misleading. It is important to recognize that these positive effects might be due to other factors than purely the use of computers. For instance, training or support from voluntary computer course instructors, teachers or supporting volunteers that increase the social relationships and participation of older people.

It is worth mentioning that D8 (leisure and activities) increased at mid-testing but decreased back to its original score at post-testing. According to Dattilo et al. [48], older people perceived doing voluntary work as a type of leisure activity that can be meaningful and enjoyable. With particular reference to Q31 and Q32 (D8) in the OPQOL questionnaire, their mid-testing scores increased a lot compared to the pre-testing scores. As indicated in the semi-structured interviews, most of the participants were more interested in helping others who needed to talk to someone. They wanted to play the role of call recipient. The use of Tangible Cup was seen as voluntary work, which made them feel more involved in things around them (Q31) and gave them a role in their life (Q32). They therefore scored higher in D8 (leisure and activities) at the beginning of the study as they felt more excited about this voluntary work. As the study progressed, they found that the other participants did not really need their help, and they started to feel less passionate about their role as volunteer. Hence, the D8 (leisure and activities) scores, i.e., Q31 and Q32 dropped drastically at post-testing.

### 4.3. Association between Technology Acceptance and Quality of Life Before Testing and Their Changes After Testing

The strength of correlations was interpreted according to Cohen’s classification where 0.10 to 0.29 is weak, 0.3 to 0.49 is moderate, and 0.5 to 1.0 is strong [49]. No significant correlation was observed between the participants’ technology acceptance and quality of life prior to using Tangible Cup. However, after using Tangible Cup for three months, the results of the Spearman’s rank-order correlation in SPSS indicate that some changes in the dimensions in technology acceptance are statistically significant associated with some changes in the dimensions in the OPQOL. 

In a study evaluating the effect of a telecare service on quality of life and technology acceptance among older people, Chou, Chang, Lee, Chou, and Mills [19] found that the user attitude to using the telecare service has the highest correlation with the quality of life. Our results indicated the same, with a correlation coefficient of 0.69 showing the highest correlation (Q in OPQOL—first question accessing overall quality of life with D7—attitude in TAM). When the participants were more positive to using ICT (Q17 and Q18 in TAM), they might feel more positive generally and give a higher score when they assessed their quality of life as a whole. The positive attitude should have made them use the ICT tools more often (D5—actual use, Q13 in TAM). However, that was not the case. The actual use of Tangible Cup correlated negatively with their quality of life. Our qualitative data could explain this; as most of the participants were already leading busy lives and the use of Tangible Cup neither fitted their ICT skills nor their lifestyle. 

The correlation results between D5 in OPQOL (home and neighborhood) with overall technology acceptance (TAM) (correlation coefficient = 0.62), D2 (perceived ease of use) (correlation coefficient = 0.59) and D8 (self-efficacy) (correlation coefficient = 0.54) in TAM, might indicate that the older participants who believed that they were able to use Tangible Cup perceived it as easy to use and thus scored higher in TAM, would feel better and happier in the home and neighborhood they were living in. The participants only used the Tangible Cup in their own home. The use of Tangible Cup, as a form of ICT intervention could help to make the participants feel they were getting more pleasure from home (D20 of OPQOL). Findings in a study by Christophorou et al. [50] confirmed that ICT services could contribute to enabling older people to stay active and independent while living at home. Some participants commented that the use of Tangible Cup would be suitable for those who had problems getting out of their homes, for instance, due to physical disabilities or bad weather in the winter and slippery conditions outside.

The Spearman rank-order correlation results also show that D4 in TAM (intention of use) is positively significantly correlated with the OPQOL total score (correlation coefficient = 0.63). When older people are more keen to use ICT tools (Q12 in TAM), they would probably perceive the use of ICT as contributing to a better quality of life. Likewise, when they have a better quality of life, they tend to be more positive to using ICT, especially when they believe that the two are interrelated. This supports the finding by Chou et al. [19] that older adults who have a more positive attitude to accepting and using telecare services have a better quality of life. Using the Internet to establish new contacts and maintain social relations has been found to have a great impact on older people’s quality of life [10]. The use of Tangible Cup, as a form of Internet-based ICT tool that can connect older people to other new people, has confirmed the positive impact on quality of life.

ICT as an intervention in reducing older people’s social isolation has helped older people through four mechanisms, i.e., connecting to the outside world, gaining social support, engaging in activities of interests and boosting self-confidence [40]. Via our semi-structured interview, we received positive feedback about the potential of Tangible Cup. Those whose needs can be met through using Tangible Cup, can benefit from using it via the above-mentioned mechanisms. Two of the participants mentioned that they would like to further develop a friendship after their conversation and meet up in person. When an older person is open to accepting the use of ICT, the benefits of using ICT to enhance their quality of life can be promising. 

### 4.4. Limitations

An obvious limitation of this study is the research design. A clinical randomized study could contribute to examining the effectiveness of the Tangible Cup as an intervention. By using an intervention group and a control group, we may be able to identify stronger evidence for the effects of using Tangible Cup. In terms of study duration, Tangible Cup was only used for three months and the participants agreed that such a short time could have little impact on their quality of life. Using Tangible Cup for a three-month period is seen as a one-off trial, so the generalizability of the results is limited, as suggested by Chen and Schulz [40] in their review of the effect of ICT interventions on reducing older people’s social isolation. 

OPQOL is a suitable instrument for measuring the multidimensional impacts on older people’s life as a result of a health and social intervention [51]. However, there were other factors that we had no control over, i.e., the participants’ taking holidays, participating in social activities, their state of health etc. While using Tangible Cup, the participants also used other ICT tools as well, such as their own smartphones, tablets, iPads, personal computers, and laptops throughout the study. These external factors affected the way they perceived ICT use and thus, their scores in the TAM and OPQOL questionnaires. In addition, it is worth mentioning that the OPQOL questionnaire representing eight dimensions [32] shows the complexity on how older people’s quality of life can be influenced. Thus, one might question on to what extend quality of life among older people may be influenced by the use of technology in terms of different dimension of their OPQOL.

The small sample size limits the precision of estimation, and thus reduces the chance of detecting the true effect of Tangible Cup as statistically significant [52]. We only managed to recruit 20 participants after approaching more than 100 potential participants. When we tried to recruit participants, we realized that those who actually had low ICT skills and might feel socially isolated were skeptical about joining our study. They felt uneasy about being labelled as needing help either with their ICT use or social life, and thus showed no interest in participating in our study. A study conducted by Zickuhr and Madden [53], found that most of the older adults stated that they were just simply not interested in using the Internet or email. All the participants in this study have used ICT for many years and were therefore more positive to trying out new technology. 

The participants did not accurately represent the target population, which means our sample is probably biased. Bilotta et al. [46] used the OPQOL questionnaire to predict several adverse health outcomes in older outpatients living in the community in Italy. In this study comprising a total of 210 older participants, the mean for OPQOL total score was 116.20. Another study conducted by Kojima et al. [47] to investigate the associations between baseline frailty status and subsequent changes in QOL had the mean for OPQOL at 130.82 (n = 363). The mean for the participants’ pre-testing OPQOL total score is 141.81 (n = 16, standard deviation = 13.20). The OPQOL total score before the use of Tangible Cup ranges from 119 to 167. This clearly indicates that the participants already had a very good quality of life before the intervention. The possibility of improving the participants’ quality of life was therefore lower.

The diversity of the participants’ socio-demographic backgrounds in our study is not well-represented. Previous studies have shown that socio-demographic variables are associated with older people’s use of ICT [54,55]. The participants in our study were recruited from another project that we had previously conducted in several senior centers located in Oslo. These senior centers are residential-area based and the participants who went to the same senior center therefore had similar socio-demographic backgrounds. They were all ethnic Norwegians and none of them were novice ICT users (refer ICT skills in Table 2). They had all lived in the City of Oslo for some time and access to ICT had never been a big issue for them. This recruitment approach has failed to reach the target user group.

Another limitation is that the validity of our instrument, the OPQOL questionnaire, is still unknown. The OPQOL questionnaire was developed by Bowling [32] and it has the potential to be used as an outcome measure to promote well-being and more active aging. This is in line with our aim, which is to use TUI to improve older adults’ quality of life and technology acceptance. The OPQOL questionnaire was chosen from among other QOL questionnaires for this very reason, because older people are our target group [56]. However, this questionnaire has never been used in Norway. We translated the questionnaire into Norwegian based on the guidelines developed by Beaton, Bombardier, Guillemin and Ferraz [33] and this Norwegian version of the OPQOL questionnaire was not validated before our data collection. We have now completed a study on the validation of this questionnaire using the methodology described by Hak et al. [57]. The methodology is a three-step test interview that involves participants’ self-completion of the OPQOL questionnaire, observation and cognitive interview. The data are currently being analyzed and will be presented in a forthcoming publication.

Last but not least, the Tangible Cup usability issue limited the participants’ use of the intervention. From our observations during visits to them, some of the participants did not always remember how to use Tangible Cup. When they did not use Tangible Cup correctly, certain functions did not work and they became frustrated. For instance, instead of putting the cup attachment on the search contacts coaster to find other online users, they put it on the call coaster. Although the participants were briefed about how to use Tangible Cup, they still needed guidance from time to time. In order to maximize the effects of using Tangible Cup, or other ICT tools as an intervention to address the issue of social isolation among older people, training is essential to address the special needs of these older ICT users [40]. Customized training and group activities must be provided on a regular basis to keep them motivated and help them to remember.

## 5. Conclusions

In this paper, we present a longitudinal study where 20 participants used a TUI prototype, Tangible Cup for three months. We have found that the use of Tangible Cup has improved the technology acceptance of some participants, but no statistically significant changes have been observed. Although the scores in the OPQOL questionnaire did not indicate much improvement in their quality of life, all of the participants agreed that Tangible Cup has the potential to improve the quality of life of other older people for whom TUI may be suitable. This group of older people might be physically limited and restricted in terms of mobility, not good at finding things to do, feel lonely and have low ICT literacy. Some statistically significant associations have been found between changes in technology acceptance and quality of life after using Tangible Cup. However, further investigation is required to validate them. We are currently further analyzing the qualitative data using a hermeneutic interpretation approach, and the qualitative data analysis exploring the participants’ experience of using the Tangible Cup might disclose more positive outcomes related to their quality of life. 

This study has shown the potential of TUIs in improving technology acceptance and quality of life. However, it is important to target the right group of older people. Our current study only lasted for three months, which might explain why the Tangible Cup had no observed impact on the users’ quality of life. In the future, we will include older people with low/no ICT skills, focus on the target user and allow the participants to use Tangible Cup for a longer period of time in order to understand the impact of Tangible Cup on their quality of life. 

In addition, the findings from this study have implications for both technology designers and developers, and clinicians and health managers. By showing the advantages of using TUI in the development and organization of clinical healthcare services for older people, digital health care services can consider TUI as a more intuitive user interface for older users. 

## Figures and Tables

**Table 1:** Technology acceptance model (TAM) questionnaire.

Dimension	Items	Reference
D1. Perceived usefulness	Q1. By using digital communication tools, I can have better social interactions with my friends.	[27,28,29,30,31]
	Q2. By using digital communication tools, I can have a better social life.	
	Q3. By using digital communication tools, I can make new friends.	
D2. Perceived ease of use	Q4. Interaction with digital communication tools is clear and understandable.	[27,28,29,30,31]
	Q5. Interaction with digital communication tools does not require a lot of mental effort.	
	Q6. I find digital communication tools easy to use.	
	Q7. I find it easy to learn to use digital communication tools.	
D3. Perceived enjoyment	Q8. I find it enjoyable to use digital communications tools.	[29]
	Q9. I find it exciting to use digital communications tools.	
	Q10. I find it pleasant to use digital communications tools.	
	Q11. I find it interesting to use digital communications tools.	
D4. Intention to use	Q12. I would use digital communication tools.	[30,31]
D5. Actual use	Q13. I use digital communication tools very often.	[29]
D6. Compatibility	Q14. Using digital communication tools is compatible with most aspects of my social life.	[28]
	Q15. Using digital communication tools fits my lifestyle.	
	Q16. Using digital communication tools fits well with the way I socialize with others.	
D7. Attitude	Q17. Using digital communication tools is a good idea.	[28,30,31]
	Q18. I am positive towards digital communication tools.	
D8. Self-efficacy	Q19. I feel confident about learning to use digital communication tools.	[29]
	Q20. I feel confident about using digital communication tools.	
	Q21. I have the necessary skills in using digital communication tools.	

**Table 2:** Summary of participants.

	Age	Gender	Education (Years)	ICT Skills
P1	79	Female	12	Basic
P2	74	Female	11	Basic
P3	82	Female	21	Basic
P4	77	Female	10	Basic
P5	76	Female	14	Very advanced
P6	81	Female	15	Basic
P7	82	Female	10	Advanced
P8	72	Female	12	Advanced
P9	82	Female	13	Basic
P10	81	Female	14	Basic
P11	81	Female	19	Advanced
P12	89	Male	17	Advanced
P13	77	Female	11	Advanced
P14	83	Male	14	Advanced
P15	83	Female	12	Advanced
P16	79	Female	12	Advanced
P17	77	Female	11	Advanced
P18	81	Female	8	Basic
P19	76	Female	13	Very advanced
P20	79	Female	10	Basic

ICT skills: Basic—manage to use smartphone and/or tablet with some problems; advanced—manage to use smartphone and/or tablet with minor problems; very advanced—manage to use smartphone and/or tablet without any problem.

**Table 3:** Summary of Spearman’s rank-order correlation (the correlations between the total score of older people’s quality of life (OPQOL) and all the OPQOL dimensions with the technology acceptance total score before using Tangible Cup).

Correlations		
	Spearman’s Rho	
	TAM (Total Score)	
	Correlation Coefficient	*p*-Value
OPQOL (total score)	−0.03	0.92
OPQOL _D1 (life overall)	−0.34	0.19
OPQOL _D2 (health)	0.20	0.46
OPQOL _D3 (social relationships and participation)	−0.41	0.12
OPQOL _D4 (independence, control over life, freedom)	0.05	0.86
OPQOL _D5 (home and neighborhood)	0.19	0.48
OPQOL _D6 (psychological and emotion well-being)	0.05	0.85
OPQOL _D7 (financial circumstances)	0.03	0.90
OPQOL _D8 (leisure and activities)	0.20	0.47
OPQOL _Q (First question evaluating quality of life as a whole)	−0.30	0.27

**Table 4:** Summary of TAM questionnaire scores in 7-point Likert scales in percentage.

	1 Strongly Disagree		2 Disagree		3 Somewhat Disagree		4 Neither Disagree nor Agree		5 Somewhat Agree		6 Agree		7 Strongly Agree	
	Pre	Post	Pre	Post	Pre	Post	Pre	Post	Pre	Post	Pre	Post	Pre	Post
D1. Perceived usefulness	12.50	14.58	6.25	4.17	12.5	8.33	18.75	10.42	20.83	31.25	14.58	22.92	14.58	8.33
D2. Perceived ease of use	3.13	1.56	9.38	3.13	12.5	21.88	34.38	10.94	12.50	31.25	23.44	31.25	4.69	0
D3. Perceived enjoyment	4.69	0	3.13	3.13	4.69	12.5	37.50	14.06	12.50	25.00	6.25	10.94	31.25	34.38
D4. Intention to use	0	0	0	0	0	6.25	31.25	12.50	12.50	0	31.25	43.75	25.00	37.50
D5. Actual use	0	0	0	0	6.25	0	6.25	6.25	25.00	12.50	37.50	37.50	25.00	43.75
D6. Compatibility	12.50	2.08	4.17	0	16.67	12.50	20.83	20.83	8.33	16.67	25.00	35.42	12.50	12.50
D7. Attitude	0	0	3.13	0	6.25	0	18.75	6.25	15.63	25.00	40.63	37.50	15,63	31.25
D8. Self-efficacy	0	0	2.08	0	8.33	2.08	0	2.08	25.00	27.08	45.83	50.00	18,75	18.75

**Table 5:** Summary of Spearman’s rank-order correlation (the correlations between the changes in all the OPQOL dimensions and the overall quality of life with all the TAM dimensions and overall technology acceptance after testing).

			OPQOL	OPQOL_D1	OPQOL_D2	OPQOL_D3	OPQOL_D4	OPQOL_D5	OPQOL_D6	OPQOL_D7	OPQOL_D8	OPQOL_Q
Spearman’s rho	TAM	Correlation Coefficient	0.21	−0.17	0.07	−0.31	−0.11	0.62 **	0.21	0.06	0.29	−0.02
		*p* -value	0.44	0.53	0.79	0.24	0.70	0.01	0.44	0.82	0.28	0.95
	TAM_D1	Correlation Coefficient	0.05	−0.11	0.02	−0.44	0.01	0.35	0.11	−0.04	0.22	−0.23
		*p* -value	0.86	0.69	0.93	0.09	0.98	0.18	0.68	0.88	0.42	0.40
	TAM_D2	Correlation Coefficient	0.29	−0.17	0.19	−0.04	−0.14	0.59 *	0.34	0.16	0.14	−0.17
		*p* -value	0.27	0.54	0.49	0.89	0.61	0.02	0.20	0.55	0.60	0.52
	TAM_D3	Correlation Coefficient	0.19	0.19	−0.07	−0.20	0.29	0.14	−0.15	0.20	0.26	0.42
		*p* -value	0.48	0.48	0.80	0.45	0.28	0.61	0.57	0.45	0.33	0.10
	TAM_D4	Correlation Coefficient	0.64 **	−0.07	0.36	0.33	0.25	0.29	−0.07	0.60 *	0.06	0.21
		*p* -value	0.01	0.80	0.17	0.22	0.35	0.28	0.80	0.02	0.84	0.43
	TAM_D5	Correlation Coefficient	0.36	−0.03	0.32	0.08	−0.03	0.27	0.50	0.27	0.07	−0.51 *
		*p* -value	0.18	0.91	0.23	0.76	0.91	0.32	0.05	0.31	0.79	0.04
	TAM_D6	Correlation Coefficient	0.20	0.08	0.11	−0.07	−0.05	0.24	0.44	0.11	0.27	−0.37
		*p* -value	0.46	0.76	0.68	0.79	0.85	0.37	0.09	0.69	0.32	0.16
	TAM_D7	Correlation Coefficient	0.29	0.11	−0.01	0.01	0.02	0.39	0.11	0.06	0.17	0.70 **
		*p* -value	0.27	0.68	0.98	0.98	0.94	0.14	0.70	0.83	0.54	0.00
	TAM_D8	Correlation Coefficient	0.23	−0.47	0.19	−0.01	−0.25	0.54 *	0.24	0.20	0.05	0.07
		*p*-value	0.40	0.07	0.48	0.96	0.36	0.03	0.38	0.45	0.90	0.80

*. Correlation is significant at the 0.05 level (2-tailed). **. Correlation is significant at the 0.01 level (2-tailed).
